# Accelerated cardiovascular magnetic resonance of the mouse heart using self-gated parallel imaging strategies does not compromise accuracy of structural and functional measures

**DOI:** 10.1186/1532-429X-12-43

**Published:** 2010-07-21

**Authors:** David Ratering, Christof Baltes, Carola Dörries, Markus Rudin

**Affiliations:** 1 Institute for Biomedical Engineering, University and ETH Zurich, Wolfgang-Pauli-Strasse 10, Zurich, 8093, Switzerland; 2 Institute of Physiology and Zurich Center for Integrative Human Physiology (ZIHP), University Zurich and Cardiology University Hospital Zurich, Winterthurerstrasse 190, Zürich, 8057, Switzerland; 3 Institute of Pharmacology & Toxicology, University Zurich, Winterthurerstrasse 190, Zurich, 8057, Switzerland

## Abstract

**Background:**

Self-gated dynamic cardiovascular magnetic resonance (CMR) enables non-invasive visualization of the heart and accurate assessment of cardiac function in mouse models of human disease. However, self-gated CMR requires the acquisition of large datasets to ensure accurate and artifact-free reconstruction of cardiac cines and is therefore hampered by long acquisition times putting high demands on the physiological stability of the animal. For this reason, we evaluated the feasibility of accelerating the data collection using the parallel imaging technique SENSE with respect to both anatomical definition and cardiac function quantification.

**Results:**

Findings obtained from accelerated data sets were compared to fully sampled reference data. Our results revealed only minor differences in image quality of short- and long-axis cardiac cines: small anatomical structures (papillary muscles and the aortic valve) and left-ventricular (LV) remodeling after myocardial infarction (MI) were accurately detected even for 3-fold accelerated data acquisition using a four-element phased array coil. Quantitative analysis of LV cardiac function (end-diastolic volume (EDV), end-systolic volume (ESV), stroke volume (SV), ejection fraction (EF) and LV mass) in healthy and infarcted animals revealed no substantial deviations from reference (fully sampled) data for all investigated acceleration factors with deviations ranging from 2% to 6% in healthy animals and from 2% to 8% in infarcted mice for the highest acceleration factor of 3.0. CNR calculations performed between LV myocardial wall and LV cavity revealed a maximum CNR decrease of 50% for the 3-fold accelerated data acquisition when compared to the fully-sampled acquisition.

**Conclusions:**

We have demonstrated the feasibility of accelerated self-gated retrospective CMR in mice using the parallel imaging technique SENSE. The proposed method led to considerably reduced acquisition times, while preserving high spatial resolution at sufficiently high CNR. The accuracy of measurements of both structural and functional parameters of the mouse heart was not compromised by the application of the proposed accelerated data acquisition method.

## Introduction

In view of increasing availability of genetically engineered strains, mouse models of human disease have become important tools for the study of pathophysiological mechanisms and the evaluation of novel therapies. A remarkable number of such transgenic models have been developed mimicking aspects of cardiovascular pathologies such as heart failure and myocardial infarction (MI). Phenotyping of disease models commonly includes investigation of the morphology and function of the heart, preferentially using non-invasive imaging methods such as cardiovascular magnetic resonance (CMR), computed tomography (CT) [1] and echocardiography [2]. In particular CMR has evolved as an accurate modality for the characterization of cardiac pathophysiology due to the inherent high soft-tissue contrast and the high spatial resolution of the method. On the other hand, dynamic CMR studies of the mouse heart face several challenges. Due to the small dimensions (the volume is three orders of magnitude smaller than the human heart) and the high heart and breathing rates of mice (heart rate: 400 - 550 beats per minute, breathing rate: 60 - 150 breaths per minute), imaging methods developed for humans can not be translated to CMR of small animals in a straightforward manner.

Cardiac MR images can be acquired following two different strategies: either using the prospective approach [3-9], i.e. MR data acquisition is directly triggered by the ECG signal, or using the retrospective approach [10,11], i.e., data is continuously collected during the experiment and subsequently reconstructed by using the stored respiratory and ECG information to assign the data to respiratory and/or cardiac phases.

In recent reports, self-gated cardiac imaging methods have been demonstrated to be a valuable tool for cardiac MR imaging in small rodents [12,13], since neither ECG leads nor respiratory motion sensors have to be utilized. The advantages of using self-gated retrospective CMR in small animal imaging are counterbalanced by long data acquisition times. Indeed, the acquisition of large datasets is required to ensure accurate and artifact-free reconstruction of cardiac movies. Long scan durations are associated with disadvantages, the most trivial one being low throughput and hence lack of efficiency. More fundamental are the high demands on the physiological/metabolic stability of the animal under investigation. Stable conditions must be maintained throughout the experiment, a particular challenge when studying diseased animals.

Accelerating self-gated cardiac imaging is therefore of high interest as demonstrated in clinical studies [14]. So-called parallel imaging techniques such as SENSitivity Encoding (SENSE) [15] represent an efficient strategy to reduce scan time, however, at the expense of decreased signal-to-noise (SNR) ratios. This is a particular concern in small animal imaging, which is inherently compromised by low SNR values due to the small voxel dimensions involved. In view of the high demands regarding sensitivity in rodent CMR and the inherent losses of SNR associated with accelerated data acquisition, the application of parallel imaging techniques in small animal CMR remains a challenge.

In this work the feasibility of accelerating self-gated cardiac imaging for assessing ventricular function in mice has been evaluated. In a first step, SENSE accelerated self-gated CMR has been adapted and optimized in order to assess morphological and functional parameters of the normal mouse heart. Subsequently, the method has been applied to self-gated CMR in mice subjected to experimental MI one week prior to the examination. The sensitivity of non-accelerated (fully sampled) self-gated retrospective CMR data in detecting structural and functional alterations due to MI was compared with that of accelerated acquisitions. Acceleration factors of up to 3.0 were investigated, resulting in a substantial decrease of the data acquisition time (from typically 20 to 6 min). For detailed comparison, contrast-to-noise ratios (CNRs) of myocardial wall as well as functional heart parameters such as left ventricular (LV) end-diastolic volume (EDV), end-systolic volume (ESV), stroke volume (SV), ejection fraction (EF) and LV mass have been calculated and compared between datasets derived from non-accelerated (acquisition time: 18.7 min) and accelerated scans (acquisition time: 6.2 min for three-fold acceleration).

## Materials and methods

### Animal Model and Preparation

All animal experiments were carried out in strict adherence to the Swiss law for animal protection. CMR acquisitions were performed in eight healthy and six infarcted female C57Bl/6J mice showing average body weights of 24.0 ± 2.1 g. MI was induced by permanent ligation of the left anterior descending coronary artery. The surgical intervention was performed as described in detail in literature [16,17]. The infarcted animals were measured one week after the surgical intervention.

For CMR experiments, mice were anesthetized with an initial dose of 3% isoflurane (Abbott, Cham, Switzerland) in air:O_2 _(4:1) and positioned on a cradle made of PMMA comprising a built-in warm water circuit. Animals were freely breathing and carefully positioned with the heart resting on the center of the RF receive coil. Body temperature was measured using a rectal temperature probe (MLT415, ADInstruments, Spechbach, Germany). Special care was taken to maintain body temperature at 36 ± 0.5°C. During the experiments anesthesia was maintained using a reduced level of isoflurane (1.6%). Mean heart and mean respiratory rates of the animals were estimated retrospectively form the acquired navigator signal yielding 498 ± 46 beats/min and 85 ± 16 breaths/min, respectively.

### Experimental Setup

All experiments were performed using a Bruker Pharmascan 47/16 (Bruker BioSpin MRI, Ettlingen, Germany) small animal MR system operating at 200 MHz with a gradient system capable of switching 350 mT/m in 200 μs. A volume resonator (75.4 mm inner diameter, active length = 70 mm) operating in quadrature mode was used for excitation and a four element (2 × 2) phased array surface coil (outer dimensions of one coil element: 12 × 16 mm^2^, total outer dimensions: 26 × 21 mm^2^) for signal reception. The coil elements were bent to a cylindrical surface (radius = 10 mm) with the axis parallel to the main magnetic field direction. The proximity of the four coil elements allowed large acceleration factors (up to three-fold acceleration) without considerably degrading image quality as each part of the imaging slice provided enough signal in all four coil elements.

Scout images of the heart anatomy were acquired for accurate planning of the subsequent cine cardiac scans. Black-blood short-axis images covering the entire left ventricle were acquired using the self-gating technique IntraGate (Bruker BioSpin MRI, Ettlingen, Germany), which is based on a fast low-angle shot (FLASH) [18] multislice sequence with an extra navigator echo. The following parameters were used for data acquisition: multislice gradient-echo sequence, field-of-view (FOV) = 25 × 25 mm^2^, matrix dimension = 154 × 154, spatial resolution = 162 × 162 μm^2^, 6 to 7 contiguous slices of 1.0 mm thickness, pulse angle = 10°, echo/repetition time (TE/TR) = 1.6/5.2 ms, number of repetitions (NR) = 200, total acquisition time = 18.7 min. Inflowing blood was saturated in a slice of 5.0 mm thickness parallel to the imaging stack with a saturation pulse angle of 60° and carefully placed at the inflow tract of the ventricles with a gap of 0.5 mm between saturation slice and first imaging slice. In addition, the navigator signal was derived from the saturation slice prior to the excitation of the cardiac imaging slices. Cardiac data were collected either in a non-accelerated manner (full k-space sampling) or accelerated in phase encoding direction (reduced k-space sampling). Acceleration factors (R) used were 1.5, 2.0, 2.5 and 3.0, resulting in total acquisition times of 12.4 min, 9.4 min, 7.5 min and 6.2 min, respectively.

In addition, the feasibility of accelerated data collection was investigated for cardiac cine images acquired in cardiac long axis orientation using a self-gated bright-blood sequence with the following parameters: FOV = 24 × 24 mm^2^, matrix dimension = 288 × 288, spatial resolution = 83 × 83 μm^2^, slice thickness = 1.0 mm, pulse angle = 15°, echo/repetition time (TE/TR) = 2.7/10 ms, number of repetitions (NR) = 300. The navigator slice was positioned at the same location as the imaging slice with a slice thickness of 1 mm. Heart data were acquired for acceleration factors of 1.0, 2.0 and 3.0, corresponding to scan durations of 14.4 min, 7.2 min and 4.8 min, respectively.

### Image Reconstruction

Cardiac cine images were reconstructed by using the collected navigator information within the IntraGate software to assign the acquired k-space lines to ten cardiac frames and one respiratory frame, retrospectively.

Coil sensitivity maps utilized for the SENSE reconstruction process were estimated from separate gradient echo low-resolution scans: FOV = 25 × 25 mm^2^, matrix dimension = 154 × 50, spatial resolution = 162 × 500 μm^2^, slice thickness = 1.0 mm, pulse angle = 10°, TE/TR = 1.4/4.1 ms, NR = 130, total scan time = 1.6 min. Calculation of sensitivity maps was performed by dividing the time-averaged single coil images by the corresponding reference images. To exclude regions of mere noise from the calculation process and for extrapolation of sensitivity maps by region growing, a signal mask was calculated using a noise level adapted threshold according to(1)

where *MEAN(S*_*Noise*_*) *and *SD(S*_*Noise*_*) *represent mean and standard deviation of signal originating from a region-of-interest (ROI) placed in an image region exhibiting pure noise.

As the volume resonator could only be operated in transmission mode, the magnitude of the reference image was calculated as the sum-of-squares (SOS) of the single coil images and the corresponding phase according to the method proposed by de Zwart et al. [19]. The noise covariance matrix of the coil elements was estimated from a separate scan and included in the reconstruction algorithm as proposed by Pruessmann et al. [15]. The reconstruction algorithm was performed on a separate workstation using in-house software written in IDL (RSI, Boulder, USA).

### Data Analysis

Validation and optimization of the proposed accelerated CMR acquisition and reconstruction method was first performed in the healthy animals: CNR analyses as well as the calculation of EDV, ESV, SV and EF were carried out in order to investigate the possible effects of accelerated data collection on image quality and on the accuracy of image derived cardiac parameters. Subsequently, the optimized acquisition and reconstruction parameters were utilized to investigate the sensitivity of the proposed accelerated CMR method in assessing myocardial remodeling induced by MI in six diseased animals.

Cardiac functional parameters were estimated from black-blood short-axis images of the left ventricle. In order to segment the left ventricular cavity a ROI was manually selected covering both ventricular cavity and wall. Subsequently, left ventricular volume and cavity were discriminated using a threshold based algorithm providing a binary image for each slice. The threshold was defined as *0.6 *• *MEAN(S*_*Left Ventricle*_*)*, where *MEAN(S*_*Left Ventricle*_*) *represents the average signal intensity of the whole left ventricle. The threshold value for intensity based image segmentation was optimized by comparing the LV volumes obtained from the MR data with references volumes from literature [6,7,20]. The best agreement was obtained for a factor of 0.6. Voxels with signal intensity higher than the threshold were attributed to the LV wall, voxels with signal intensity lower than the threshold to the LV cavity. Segmentation of cardiac images and calculation of EDV and ESV was performed using in-house software written in IDL.

EDV and ESV were determined in end-diastolic and end-systolic phase, respectively, by counting the voxels within the segmented left-ventricular (LV) cavity for all slices covering the entire left ventricle. End-diastole and end-systole were defined as the cine frame with maximal and minimal LV volume, respectively. Subsequently, the counted number of voxels was multiplied by the voxel volume (0.162 × 0.162 × 1 mm^3^). SV was calculated as the difference between EDV and ESV, and EF as ratio SV/EDV. For LV mass determination, the epi- and endocardial borders in the end-diastolic and end-systolic frames have been outlined manually in order to segment the LV myocardium. LV mass was estimated by multiplying the number of voxels contained in the LV myocardium by the voxel volume and the specific gravity of 1.05 g/cm^3 ^[21]. LV mass calculation was performed for both healthy and infarcted animals for acceleration factors R = 1.0 and R = 3.0. In addition, CNR values were computed for the LV myocardial wall for end-systolic and end-diastolic phase. Being aware that the noise distribution in SENSE reconstructed images varies spatially, we evaluated CNR between the LV myocardial wall and the LV cavity by just focusing on these structures of interest in order to assess the ability to discriminate wall tissue from the ventricular cavity in images reconstructed from accelerated datasets. This is essential for accurate assessment of LV function. Accordingly, *CNR*_*Wall-Cavity *_was calculated as(2)

where *S*_*Myocardial Wall*_, *S*_*Ventricular Cavity*_, *SD(S*_*Myocardial Wall*_*) *and *SD(S*_*Ventricular Cavity*_*) *are defined as the signal of the myocardial wall, the signal of the ventricular cavity and the standard deviation of the myocardial wall and ventricular cavity signal, respectively. All CNR analyses of this study were performed for the accelerated self-gated retrospective black-blood measurements using in-house software written in IDL. CNR calculations were performed for all acceleration factors under investigation according to the threshold based segmentation algorithm described previously.

Finally, the effect of the accelerated data collection on the functional cardiac parameters (*P*_*LV*_) was investigated by estimating their deviation (Δ*P*_*LV*_) from the reference value obtained from fully sampled reconstructed [22] images. The mean deviation of the functional parameter of interest was calculated over all investigated animals (*N*) according to:(3)

where *P*_*LV; R,i *_is defined as the LV parameter for acceleration factor R (R = 1.5, 2.0, 2.5, 3.0) and *P*_*LV; R = 1.0,i *_as the LV parameter for acceleration factor 1.0 for each animal i. Calculations according to Eq. [3] were performed for the LV functional parameters EDV, ESV, SV, EF and LV mass.

Finally, in order to assess reproducibility of our proposed method, inter- and intra-observer variability measurements were performed by two independent observers as well as twice by one observer for all investigated cardiac functional parameters for R = 1.0 and R = 3.0.

## Results

### Impact of accelerated CMR on the anatomical definition of the mouse heart

**Figure 1:**
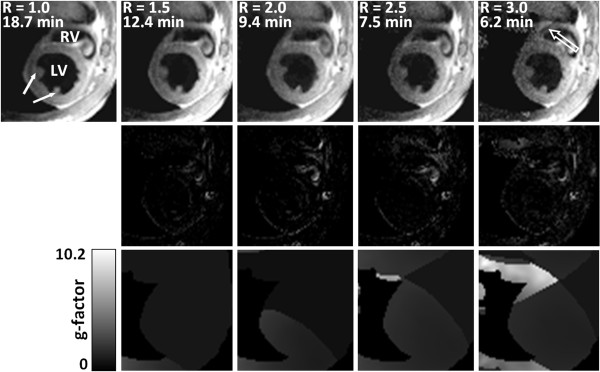
**Mid-ventricular short-axis views of a mouse heart for different acceleration factors**. Top row: representative mid-ventricular black-blood short-axis views of a healthy mouse heart (left (LV) and right ventricle (RV)) for all investigated acceleration factors R: R-values and corresponding acquisition times are given in the upper left corners. Solid white arrows indicate the papillary muscles which are accurately depicted even for an acceleration factor of R = 3.0. The open white arrow indicates a reconstruction artifact for R = 3.0 due to elevated local g-factor values. Middle row: corresponding difference images calculated between fully sampled image (R = 1.0) and images obtained from accelerated acquisitions. Bottom row: corresponding g-factor maps.

High-resolution short-axis cardiac black-blood images depicting the heart anatomy with high anatomical definition (in-plane resolution of 162 × 162 μm^2^) and excellent contrast between LV myocardial wall and LV cavity were obtained for all investigated acceleration factors. Representative images of a mid-ventricular short-axis view of the left and right ventricle of a healthy mouse heart are shown in Figure 1 for a fully sampled acquisition and acquisitions accelerated using factors R = 1.5, 2.0, 2.5 and 3.0. The total data acquisition time for the fully sampled data set amounted to 18.7 min. Difference images between accelerated and non-accelerated reference acquisitions (Figure 1) show only minor deviations. Upon increasing the R-value noise contributions in the images increase as expected [15]. Indeed, local noise enhancement, which is known to be a characteristic property of SENSE reconstructed images, was most notably observed for images acquired with an acceleration factor of R = 3.0. Noise enhancement due to SENSE is not constant across the image but influenced by geometrical factors of the coil. To analyze regions of high noise enhancement, so-called geometry factor (g-factor) maps were computed (Figure 1). An example of local noise enhancement leading to an image artifact is shown in Figure 1 (open white arrow) for an acceleration factor of R = 3.0. In the high g-value area (> 10) the regularization of the SENSE reconstruction process produced an image artifact that could potentially be interpreted as anatomical structure.

**Figure 2:**
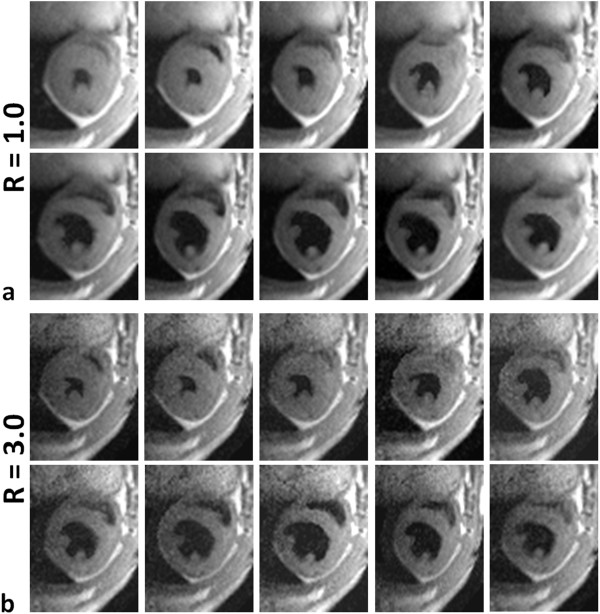
**Time series of cardiac black-blood short-axis views illustrating a whole cardiac cycle for a mid-ventricular slice**. Non-accelerated and accelerated images were acquired in the same healthy animal for acceleration factors of R = 1.0 **(a) **and R = 3.0 **(b)**. Good contrast between LV wall and LV cavity was achieved even for R = 3.0.

Despite increased noise levels for large R-values, acceleration did not compromise the detectability of small anatomical heart structures such as the papillary muscles (solid white arrows in Figure 1): the inherent CNR was sufficiently high to warrant the detectability of these structures even for acceleration factor R = 3.0. Figure 2 shows representative time series of cardiac black-blood short-axis views illustrating a whole cardiac cycle for a mid-ventricular slice. Non-accelerated (R = 1.0, Figure 2a) and accelerated (R = 3.0, Figure 2b) short-axis views were acquired in the same healthy animal: adequate image quality was preserved for the accelerated short-axis images and CNR was high enough to achieve good contrast between LV wall and LV cavity even for R = 3.0.

**Figure 3:**
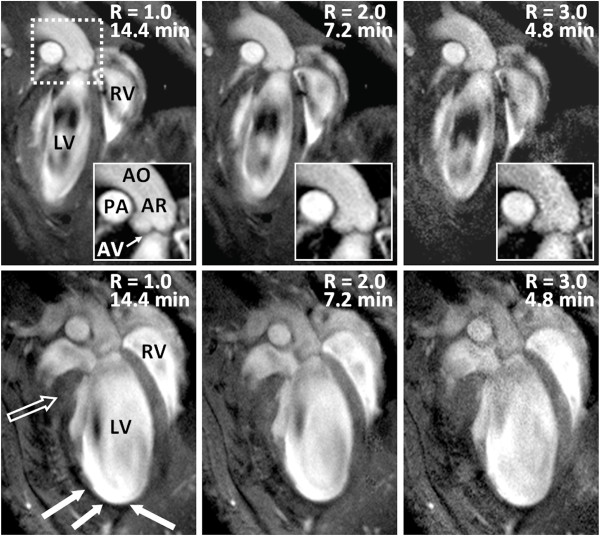
**Representative bright-blood long-axis viewes (left (LV) and right ventricle (RV)) of a healthy (top row) and infarcted (bottom row) animal for different acceleration factors R**. Inserts corresponding to the dashed white box in the top row show the magnified left ventricular outflow tract with pulmonary artery (PA), aorta (AO), aortic root (AR) and aortic valve (AV). The contrast in the inserts was equally modified in order to better visualize the AV, which can be clearly identified even for an acceleration factor of R = 3.0. The long-axis views in the bottom row clearly illustrate the enlargement of the left ventricle due to LV remodeling after myocardial infarction (MI): the open arrow shows LV wall thickening whereas the white arrows indicate LV wall thinning. LV remodeling is accurately detected even for R = 3.0.

In order to demonstrate the feasibility of accelerated data collection for long-axis views of the mouse heart, images were acquired in long-axis orientation for R-values of 1.0, 2.0 and 3.0 in one healthy and in one diseased animal. A representative long-axis view of a healthy animal displays right and left ventricles, the aortic root (AR) and valve (AV) as well as the pulmonary artery (PA) for all investigated acceleration factors R. In particular, the good definition of the AV is remarkable as illustrated by the magnified views (inserts Figure 3). Even for an acceleration factor of R = 3.0, CNR was high enough to discriminate the aortic valves from the surrounding blood. The acquisition time for the fully sampled data set amounted to 14.4 min. The bottom row of Figure 3 illustrates a long-axis view of an animal which underwent MI one week earlier. Dilation of the left ventricle due to LV remodeling is clearly visible. Furthermore, thickening of the unaffected myocardial wall (open arrow, Figure 3) and thinning of infarcted tissue (white arrows, Figure 3) are accurately reproduced even in images acquired with an acceleration factor of R = 3.0.

**Figure 4:**
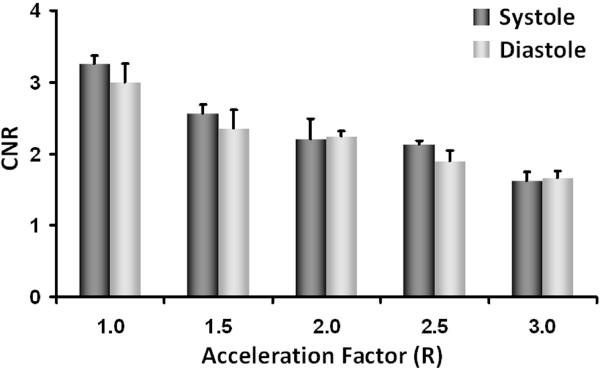
***CNR*_*Wall-Cavity *_values for end-systolic and end-diastolic heart phase calculated according to Eq. [2] in eight healthy animals.** Values are given as mean ± SD for all investigated acceleration factors R.

In order to investigate the effects of accelerated data collection on image quality, a quantitative analysis in terms of CNR was performed in all eight healthy animals for the LV myocardial wall and cavity. CNR was calculated for end-systolic and end-diastolic phase according to Eq. [2]. The mean absolute CNR values are indicated in Figure 4 for both end-systolic and end-diastolic cardiac phase. For end-systole CNR decreased from a value of 3.25 for R = 1.0 to a value of 1.62 for R = 3.0. Similar results were found for end-diastole, CNR decreased from a value of 3.01 for R = 1.0 to a value of 1.65 for R = 3.0. These CNR decreases amount to a maximum average decrease in CNR of 50% during end-systole and of 45% during end-diastole for the highest acceleration factor of R = 3.0.

### Impact of accelerated CMR on quantitative functional ventricular parameters

Quantitative analysis of LV cardiac function (EDV, ESV, SV, EF) was carried out in eight healthy animals. The analyzed parameters were calculated for each animal and for all investigated acceleration factors (Table 1). Deviations of mean values derived from accelerated data sets (R = 1.5, 2.0, 2.5 and 3.0) from values derived from the reference data (R = 1.0) were found to be negligible (< 2%) for all functional parameters and all investigated acceleration factors. Good agreement between non-accelerated and accelerated data sets was also found for LV mass determination of healthy and infarcted animals: absolute mean values of 93.6 ± 5.7 mg (healthy animals) and 152.9 ± 6.7 mg (infarcted animals) for an acceleration factor of R = 1.0 and 94.1 ± 8.0 mg (healthy animals) and 157.6 ± 6.4 mg (infarcted animals) for R = 3.0 were obtained. In addition, mean deviations from reference for the LV mass values were calculated for R = 3.0 according to Eq. [3] and were found to be 3.3 ± 2.5% and 3.5 ± 2.6% for healthy and infarcted animals, respectively.

**Table 1:** Quantitative assessment of LV function in eight healthy animals: EDV, ESV, SV and EF parameters were derived for all investigated acceleration factors R.

	*R = 1.0*	*R = 1.5*	*R = 2.0*	*R = 2.5*	*R = 3.0*
***EDV [μl]***	44.2 ± 2.0	44.3 ± 1.8	45.2 ± 2.2	45.0 ± 1.9	44.2 ± 2.5

***ESV [μl]***	12.1 ± 0.8	12.2 ± 0.9	12.4 ± 1.0	12.4 ± 1.1	12.2 ± 1.3

***SV [μl]***	32.2 ± 1.5	32.1 ± 1.2	32.6 ± 1.4	32.6 ± 1.7	32.0 ± 1.6

***EF [%]***	72.8 ± 1.1	72.5 ± 1.3	72.6 ± 1.4	72.5 ± 2.2	72.5 ± 1.9

All values are given as mean ± SD.

**Figure 5:**
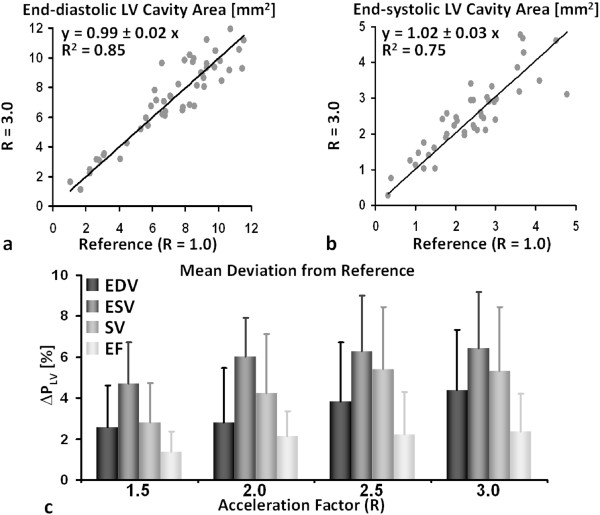
**Correlation of LV cavity areas of eight healthy animals for end-diastolic phase (a) and end-systolic phase (b) obtained from reference acquisitions (R = 1.0) and acquisitions accelerated by a factor of R = 3.0**. The slope of the linear fits and the R^2 ^values indicate the degree of correlation between accelerated and non-accelerated CMR acquisition. **(c) **Mean deviation of LV parameters (Δ*P*_*LV*_) of end-diastolic volume (EDV), end-systolic volume (ESV), stroke volume (SV) and ejection fraction (EF) from the reference values (LV parameters obtained for R = 1.0) calculated according to Eq. [3] for all acceleration factors R over all healthy animals (N = 8).

In order to carefully evaluate the accuracy of morphometric analyses, end-diastolic and end-systolic LV cross-sectional areas of accelerated acquisitions were correlated for each slice of the multislice image dataset to the corresponding values of the non-accelerated reference scan. Linear regression analysis was carried out for each investigated acceleration factor and confirmed the good agreement between non-accelerated and accelerated data. For R = 3.0 correlation coefficients of R^2 ^= 0.85 and 0.75 have been obtained for LV cavity areas during end-diastole and end-systole, respectively, with corresponding slopes of regression line of 0.99 ± 0.02 and 1.02 ± 0.03 (Figure 5a, b). Similar correlation coefficients could also be found for the other acceleration factors: for LV areas during end-diastole R^2 ^values of 0.88, 0.86 and 0.85 with corresponding slopes of 0.99 ± 0.02, 1.01 ± 0.01 and 1.02 ± 0.02 were obtained for acceleration factors of R = 1.5, 2.0 and 2.5, respectively. For the LV cavity areas during end-systole the corresponding R^2 ^values were 0.82, 0.74 and 0.75 with slopes of 0.99 ± 0.01, 0.98 ± 0.02 and 1.02 ± 0.01 for acceleration factors of R = 1.5, 2.0 and 2.5, respectively. In addition, no systematic bias for EDV and ESV values was observed when analyzing the corresponding correlation plots.

Finally, the mean deviation of the various functional parameters (Δ*P*_*LV*_) from the reference values obtained from the R = 1.0 reference datasets was analyzed for all investigated acceleration factors according to Eq. [3]. Using the absolute value of differences, i.e. Eq. [3], allowed to avoid possible averaging effects when calculating the mean values of the different LV parameters. The results are summarized in Figure 5c: the maximum mean deviation Δ*P*_*LV *_of 6% was found for the ESV for an acceleration factor of R = 3.0. All other mean deviations estimated for EDV, SV and EF were found to be < 5% demonstrating that acceleration of data acquisition did not significantly compromise the accuracy of morphometric measurements.

Finally, good reproducibility of our proposed accelerated acquisition method was found for all investigated LV functional parameters for R = 1.0 and R = 3.0. The results obtained from inter- and intra-observer variability measurements are summarized in Table 2.

**Table 2:** Inter- and intra-observer variability assessment in eight healthy animals for all investigated LV parameters: EDV, ESV, SV, EF and mass.

	*Inter-observer variability [%]*		*Intra-observer variability [%]*	
	***R = 1.0***	***R = 3.0***	***R = 1.0***	***R = 3.0***

***EDV***	2.9 ± 1.8	3.4 ± 1.7	2.3 ± 1.9	2.9 ± 2.5

***ESV***	3.3 ± 3.9	4.1 ± 3.1	3.1 ± 2.4	3.9 ± 2.9

***SV***	4.5 ± 2.2	5.3 ± 1.9	3.9 ± 2.5	4.9 ± 2.6

***EF***	3.3 ± 1.6	3.9 ± 2.4	2.8 ± 1.9	3.4 ± 2.4

***Mass***	4.3 ± 2.6	4.4 ± 4.1	4.1 ± 2.1	4.8 ± 3.9

Measurements were performed for acceleration factors R = 1.0 and R = 3.0. All values are given as mean ± SD.

### Sensitivity of accelerated CMR in assessing myocardial remodeling after MI

**Figure 6:**
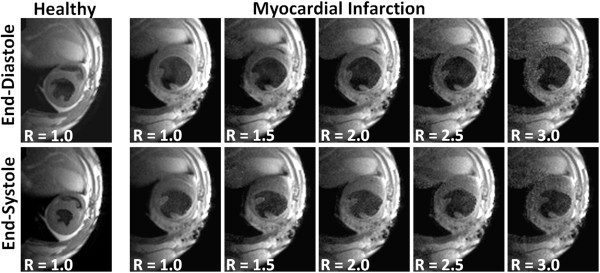
**Black-blood short-axis views of a mid-ventricular slice before and after MI for end-diastolic phase (top row) and end-systolic phase (bottom row) in the same animal**. Even for an acceleration factor of R = 3.0, LV enlargement is clearly visible after MI when compared to the LV dimensions before MI. Good contrast was achieved between LV myocardial wall and LV cavity for all investigated acceleration factors R.

The accelerated self-gated retrospective black-blood CMR method was applied to evaluate the accuracy in assessing myocardial remodeling after experimental MI. Figure 6 illustrates a representative example of a transverse mid-ventricular slice acquired in an infarcted animal in short-axis orientation for both end-diastolic and end-systolic phase of the cardiac cycle for R = 1.0, 1.5, 2.0, 2.5 and 3.0. Increasing the R-value increases the noise contributions in the images, but good image quality and high contrast between LV wall and LV cavity were preserved even for an acceleration factor of R = 3.0. Comparison with end-diastolic and end-systolic images of the same animal recorded prior to MI revealed a marked increase in left LV dimensions in both cardiac phases. This translates into significantly impaired LV functional parameters.

**Figure 7:**
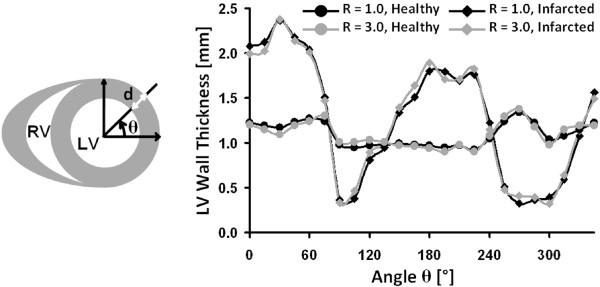
**Wall thickening and wall thinning in the infarcted animal is clearly visible from the LV wall thickness profiles for both non-accelerated (R = 1.0) and accelerated (R = 3.0) measurements**. Left: drawing illustrating the coordinate system taken as reference for the measurement of the angle θ at which LV wall thickness (d) was measured. Measurements were performed in steps of 15°. Right: LV wall thickness was measured for a selected slice in a healthy (circles) and an infarcted animal (diamonds, MI) for acceleration factors of R = 1.0 (black lines) and R = 3.0 (gray lines).

In order to demonstrate the ability of the proposed accelerated acquisition method to detect myocardial remodeling after MI, the LV myocardial wall thickness of one infarcted animal was measured for a selected slice acquired with acceleration factors R = 1.0 and R = 3.0. Wall thickness was measured every 15°. For comparison, LV wall thickness measurements were performed in a healthy animal for a slice taken approximately at the same position. The results are reported in Figure 7, where the wall thickness profile obtained for R = 3.0 shows only minor deviations from the profile obtained for R = 1.0. LV wall thickening and thinning in the infarcted animal is clearly visible, whereas the wall thickness in the healthy animal remains relatively constant.

Table 3 lists the mean values of EDV, ESV, SV and EF calculated for the infarcted mice for all investigated acceleration factors R. The values confirm the LV dilation and contractile dysfunction of the mouse heart as a result of MI. EDV and ESV in infarcted animals are significantly larger than those observed in healthy mice with EDV increasing from ~44 μl to ~86 μl, and ESV from ~12 μl to ~68 μl. The reduced amount of blood ejected results in decreases of both SV (from ~32 μl in the healthy animals to ~17 μl in the infarcted animals) and EF (from ~72% in the healthy animals to ~22% in the infarcted animals). The values in Table 3 furthermore confirm the good agreement of cardiac functional parameters derived from accelerated acquisitions when compared to the reference values (R = 1.0). For SV and EF the maximum deviations from the reference values upon acceleration amounted to 6.0% and 5.0%, respectively, for R = 3.0. For the average EDV and ESV values the corresponding deviations were found to be ≤ 2.5%.

**Table 3:** Quantitative assessment of LV function in six animals subjected to MI: EDV, ESV, SV and EF parameters were derived for all investigated acceleration factors R.

	*R = 1.0*	*R = 1.5*	*R = 2.0*	*R = 2.5*	*R = 3.0*
***EDV [μl]***	86.4 ± 3.8	85.9 ± 4.7	84.2 ± 7.3	87.4 ± 4.9	88.2 ± 5.5

***ESV [μl]***	68.1 ± 3.9	68.1 ± 5.4	66.9 ± 6.9	69.1 ± 5.4	68.9 ± 5.5

***SV [μl]***	17.9 ± 1.2	17.8 ± 1.1	17.4 ± 1.5	18.3 ± 0.9	19.0 ± 2.5

***EF [%]***	22.8 ± 1.6	22.9 ± 2.2	22.9 ± 2.1	23.1 ± 1.9	24.0 ± 2.8

All values are given as mean ± SD.

**Figure 8:**
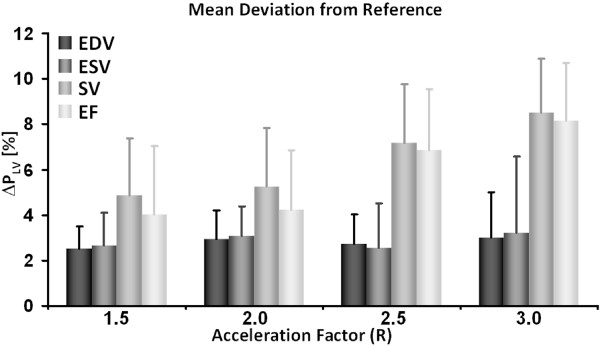
**Mean deviation of LV parameters from reference values calculated for the infarcted animals**. Mean deviation of LV parameters (Δ*P*_*LV*_) of end-diastolic volume (EDV), end-systolic volume (ESV), stroke volume (SV) and ejection fraction (EF) from the reference values (LV parameters obtained for R = 1.0) calculated according to Eq. [3] for all acceleration factors R over all infarcted animals (N = 6).

The mean deviations of the functional parameters (Δ*P*_*LV*_, Eq. [3]) are summarized in Figure 8. The maximum mean deviations Δ*P*_*LV *_were found for the SV and EF for an acceleration factor of R = 3.0 amounting to 8.4% and 8.1%, respectively. The maximum mean deviations estimated for EDV and ESV were found to be 2.9% and 3.2%, respectively, for an acceleration factor of R = 3.0. The higher Δ*P*_*LV *_values for SV and EF are due to the fact, that relatively high EDV (84 - 88 μl) and ESV (66 - 68 μl) values were obtained from the LV function assessment, whereas SV (17 - 19 μl) and EF (22% - 24%) values, which were derived from the difference of EDV and ESV and the ratio of SV and EDV, resulted to be relatively small and hence led to comparatively large deviations Δ*P*_*LV*_.

In summary, only minor deviations from the reference values (LV parameters obtained for R = 1.0) could be found for all calculated cardiac functional parameters.

## Discussion

Self-gated CMR has been shown to yield accurate structural and functional measures of the mouse heart [12,13]. Yet, the approach suffers from long acquisition times in order to acquire a large enough data basis for reconstructing the different phases of the cardiac cycle. Our study shows that the self-gated CMR data acquisition scheme can be successfully accelerated yielding CNR values that are sufficient for an accurate assessment of the dynamic LV morphology in mice. Significant shortening of acquisition times by factors between 1.5 and 3.0 have been achieved using the parallel CMR approach SENSE, while still preserving good image quality. Indeed, the results of this study demonstrate that 3-fold acceleration of multi-slice CMR of the mouse heart can be successfully achieved (R = 3.0) in combination with a four-element phased array coil. This translates into a scan time reduction from 18.7 min (R = 1.0) to 6.2 min (R = 3.0) for the multi-slice black-blood short-axis view and from 14.4 min (R = 1.0) to 4.8 min (R = 3.0) for the long-axis bright-blood view of the mouse heart. Similar scan time reductions have been reported by J.E. Schneider and coworkers [23], which have investigated the effects of TGRAPPA [24] reconstruction on cardiac function assessment using a four-element (4 × 1) RF coil array. However, in comparison to our study, measurements have been carried out in rats at a field strength of 9.4 T using an ECG-triggered and respiratory gated sequence. In addition, the accelerated cardiac data sets were simulated offline form the acquired fully sampled acquisitions. It is shown, that measurement times could be decreased from 11 min (R = 1.0) to approximately 3 min for an acceleration factor of R = 4.0 without severely compromising image quality and cardiac function assessment.

Even though CNR decreases due to inherent sensitivity losses of SENSE reconstructed data, good quality black-blood and bright-blood images of the mouse heart could be obtained for all acceleration factors. Indeed, despite reductions in *CNR*_*Wall-Cavity *_calculated between LV myocardial wall and LV cavity (45 - 50% for R = 3.0, Figure 4), high definition short- and long-axis views of the mouse heart have been obtained in both healthy and diseased animals. This is demonstrated by the excellent definition of the aortic valve in the bright-blood images (Figure 3), the dynamics of which can be monitored throughout the cardiac cycle. Similarly, for all R values contrast between LV myocardial wall and cavity in black-blood images was sufficient to allow for unambiguous delineation of epi- and endocardial borders, an important prerequisite for accurate determination of functional cardiac parameters. Small anatomical structures, such as the papillary muscles could be depicted with excellent anatomical detail even for an acceleration factor of R = 3.0 (Figures 1, 2 and 3). Furthermore, the proposed accelerated acquisition method was sensitive enough to accurately detect myocardial remodeling after MI, such as LV enlargement and LV wall thickening/thinning, even for an acceleration factor of R = 3.0 (Figures 3, 6 and 7).

LV functional parameters derived from the acquired data of both healthy and infarcted animals were found to be comparable with values already reported in literature [1,6,7,11,20]. The good agreement between non-accelerated and accelerated acquisitions is documented by the correlation of cross-sectional LV cavity areas (Figure 5 a, b) and the small mean deviations of LV functional parameters (Δ*P*_*LV*_) derived from accelerated acquisitions from values obtained from fully-sampled data (Figures 5c and 8). Thus, the quantitative analysis of cardiac function revealed that accelerating data acquisition did not substantially reduce the precision in assessing LV functional parameters in both healthy and diseased animals. In addition, reproducibility of the proposed accelerated data acquisition method in assessing LV function of the mouse heart was demonstrated from findings obtained from inter- and intra-observer variability measurements (Table 2).

Increasing spatial in-plane resolution for 2D imaging or performing four-dimensional CMR (4D-CMR, 3 spatial and 1 temporal dimension) allowing for high spatial resolution in all three dimensions would have beneficial effects on the depiction of the cardiac anatomy and the derivation of cardiac functional parameters by minimizing partial volume effects. On the other hand, increasing spatial resolution has the drawback of increased scan durations, making the application of the proposed accelerated acquisition method even more attractive. In plane spatial resolution in the current study was 83 × 83 μm^2 ^for bright-blood and 162 × 162 μm^2 ^for black-blood images. While resolution can be improved in principle, there are practical limitations. Reducing the voxel size will decrease SNR and consequently CNR, making unambiguous identification of tissue borders difficult. A second potentially limiting factor is the size of the acquired four-dimensional dataset, which will translate into long processing times.

The sensitivity issue can be addressed by applying the SENSE based accelerated data acquisition method to 4D self-gated retrospective CMR. Furthermore, the spatial encoding efficiency of a receiver array is enhanced for 3D imaging since acceleration can be done in both phase encoding directions [25]. Finally, higher acceleration factors and/or better image quality of SENSE reconstructed cardiac images might be achievable by increasing the number of coil elements of the receiver array or by optimizing the coil geometry and hence reducing local noise enhancement in the region of the heart [26].

## Conclusion

In conclusion, we have demonstrated the feasibility of accelerating self-gated retrospective CMR in mice using the parallel imaging technique SENSE in combination with a four-element phased array surface coil for signal detection. The accuracy of the accelerated acquisition allows identification of small anatomical heart structures such as the papillary muscles and the aortic valve as well as a reliable quantification of left ventricular function. Accelerating data collection using SENSE led to considerably reduced acquisition times (up to factor 3.0), while preserving high spatial resolution at sufficiently high CNR, a prerequisite for accurate calculation of cardiac functional parameters. Finally, quantitative analysis of cardiac function and assessment of myocardial remodeling in infarcted animals was successfully performed demonstrating the feasibility of the developed method to follow disease progression and therapy.

## Competing interests

The authors declare that they have no competing interests.

## Authors' contributions

**DR **and **CB **were responsible for study design, implementation of the accelerated self-gated CMR method, for data acquisition and analysis and for manuscript preparation. **CD **was responsible for inducing myocardial infarction in the investigated animals. **MR **has supervised the project and contributed to the manuscript. All authors have read and approved the final version of the manuscript.
